# Long non‐coding RNAs in cutaneous biology and proliferative skin diseases: Advances and perspectives

**DOI:** 10.1111/cpr.12698

**Published:** 2019-10-06

**Authors:** Lipeng Tang, Yongxin Liang, Hesong Xie, Xiaozhi Yang, Guangjuan Zheng

**Affiliations:** ^1^ Department of Pharmacology of Traditional Chinese Medicine The Second Affiliated Hospital of Guangzhou University of Chinese Medicine Guangzhou China; ^2^ School of Bioscience and Bio‐pharmaceutics Guangdong Pharmaceutical University Guangzhou China; ^3^ Guangzhou Virotech Pharmaceutical Co., Ltd Guangzhou China; ^4^ Department of Pathology The Second Affiliated Hospital of Guangzhou University of Chinese Medicine Guangzhou China

**Keywords:** cutaneous squamous cell carcinoma, keratinocyte differentiation, long non‐coding RNAs, melanoma, psoriasis, wound healing

## Abstract

Advances in transcriptome sequencing have revealed that the genome fraction largely encodes for thousands of non‐coding RNAs. Long non‐coding RNAs (lncRNAs), which are a class of non–protein‐coding RNAs longer than approximately 200 nucleotides in length, are emerging as key epigenetic regulators of gene expression recently. Intensive studies have characterized their crucial roles in cutaneous biology and diseases. In this review, we address the promotive or suppressive effects of lncRNAs on cutaneous physiological processes. Then, we focus on the pathogenic role of dysfunctional lncRNAs in a variety of proliferative skin diseases. These evidences suggest that lncRNAs have indispensable roles in the processes of skin biology. Additionally, lncRNAs might be promising biomarkers and therapeutic targets for cutaneous disorders.

## 1 INTRODUCTION

Skin, which is the largest organ in the human body, accounts for almost 15% of the total adult bodyweight. It protects the organism from environmental stresses, such as dehydration, irradiation, mechanical trauma and pathogenic infection. The essential functions of skin rely on its stratification, the adhesion level between layers and different cell types and various signals that fine‐tune gene expression which maintains skin homeostasis. Previously, lots of researches have described the involvement of coding genes in skin development and diseases.^1^, ^2^ However, the regulatory roles of non‐coding genes in skin biology and diseases still need to be further elucidated.

Advances in high‐throughput deep sequencing of the transcriptome and the ENCODE project over the last two decades have demonstrated that only approximately 2% of the genome codes for proteins, while the largest genome fraction encodes for thousands of non‐coding RNAs.^3^, ^4^ These results indicate that non‐coding RNAs, which were considered as “transcriptional noises,” play a critical role in the gene control across all kingdoms of life.

According to the length of transcripts, modulatory non‐coding RNAs can be categorized into two distinct classes: small (such as microRNAs) and long non‐coding RNAs (lncRNAs).^5^, ^6^ LncRNAs are a large and diverse class of non‐coding RNA molecules defined as non–protein‐coding RNAs that are >200 nucleotides in length1.^7^, ^8^ Previous studies have certified that lncRNAs almost participated in all aspects of biological behaviours, such as epigenetics,^9^, ^10^ transcription^11^, ^12^ and post‐transcription.^13^, ^14^, ^15^ Additionally, they are proved to be involved in biological processes both physiologically^16^, ^17^, ^18^ and pathologically.^19^, ^20^

Recently, researchers have taken advantage of microarray and high‐throughput deep sequencing to further explore the regulatory effects of lncRNAs in the field of cutaneous research.^21^ Emerging evidence indicates that lncRNAs not only play an essential role in the biology of skin development but also in the pathology of cutaneous proliferation‐related diseases.^22^, ^23^ In this review, we summarize an overview of current knowledge regarding the roles of lncRNAs in skin biology and disease, discussing the challenges and the potential clinical applications that they offer.

## 2 LncRNA CLASSIFICATION AND REGULATORY MECHANISMS

**Figure 1:**
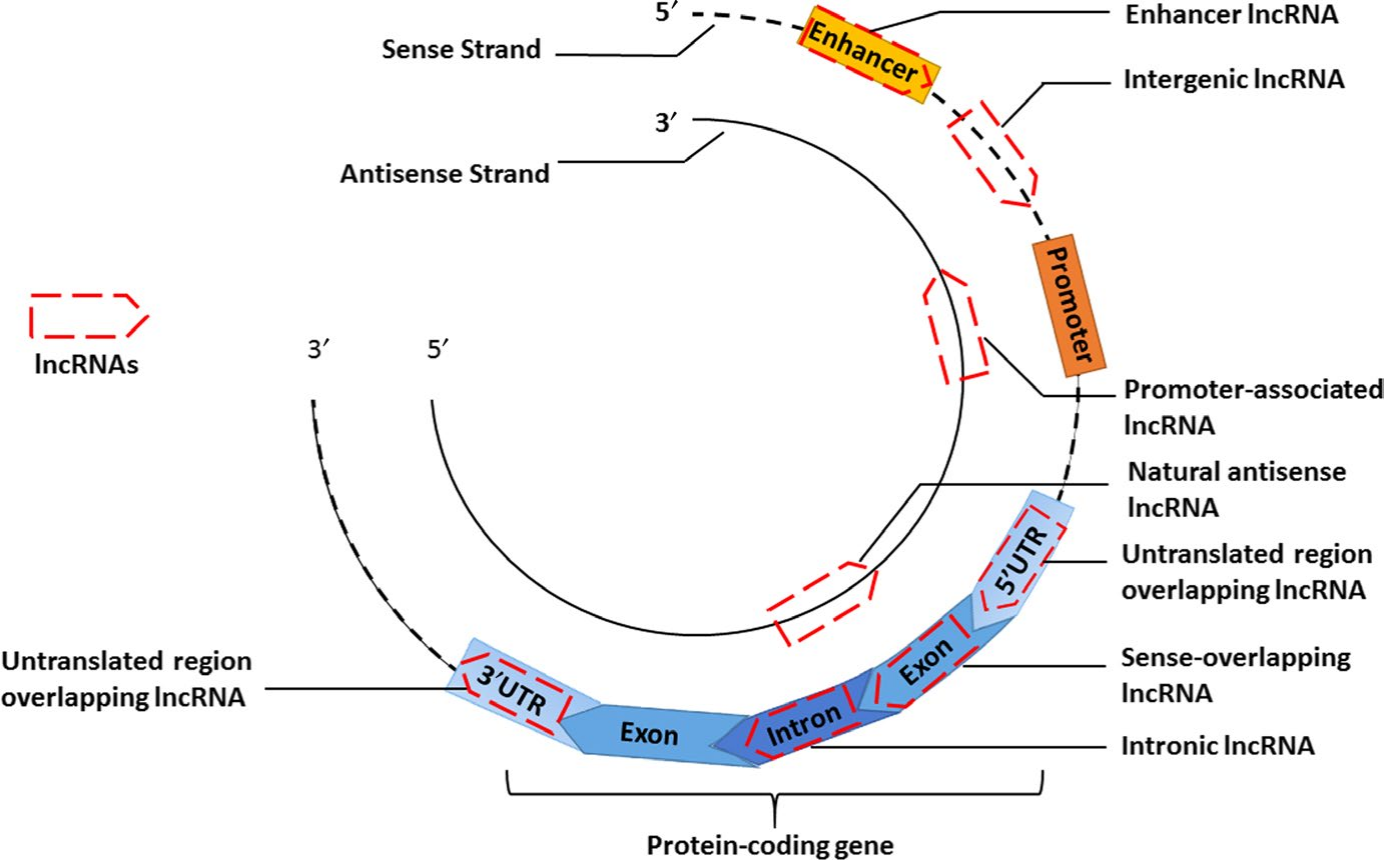
The classification of long non‐coding RNA. Long non‐coding RNAs (lncRNAs) can be grouped according to their transcribed genomic positions

LncRNAs can be grouped according to the genomic location from which they are transcribed^5^, ^20^, ^24^ (Figure 1). For example, enhancer lncRNAs originate from enhancer regions, and promoter‐associated lncRNAs are transcribed in the opposite direction to the protein‐coding transcript from regions in close proximity to a promoter. In addition, intergenic lncRNAs (lincRNAs) are transcribed from non‐coding DNA sequences located between protein‐coding genes, whereas intronic lncRNAs are transcribed from the introns of protein‐coding genes. Sense lncRNAs and natural antisense lncRNAs are transcribed from the sense and antisense strands of protein‐coding genes, respectively. Additionally, sense lncRNAs and natural antisense lncRNAs can overlap with one or several introns and/or exons of the sense sequence. Untranslated region (UTR) overlapping lncRNAs are transcribed from DNA sequences overlapping the 3′UTR or 5′UTR region of a protein‐coding gene in the sense strand (Figure 1).

LncRNAs modulate gene expression by interacting with DNA, RNA or proteins via structural interactions and/or complementary base pairing. They can regulate gene expression in multiple ways, including epigenetic modification, transcription, post‐transcription, translation and post‐translation. For example, epigenetic control by lncRNAs, such as X inactivation,^25^ genomic imprinting,^26^ mediation of mRNA stability enhancement or decay^27^ and neutralization of miRNAs,^28^ has been reported. At the transcriptional level, lncRNAs play activator roles in enhancing or inhibiting protein‐coding gene transcription. These lncRNAs are usually synthesized at enhancers. They influence the activity of enhancers or recruit protein complexes to enhancers, acting as cofactors that help remodel chromatin architecture and enhance kinase activity.^29^ At the post‐transcriptional level, lncRNAs regulate diverse processes, such as transport, translation, splicing or decay of mRNA and neutralization of miRNAs.^30^, ^31^, ^32^

Recently, growing evidence indicates that lncRNAs exert important effects on the biological processes of cutaneous development as well as on the pathogenesis of skin diseases via multiple ways mentioned above.

## 3 THE INDISPENSABLE ROLES OF LncRNAS IN CUTANEOUS BIOLOGY

**Figure 2:**
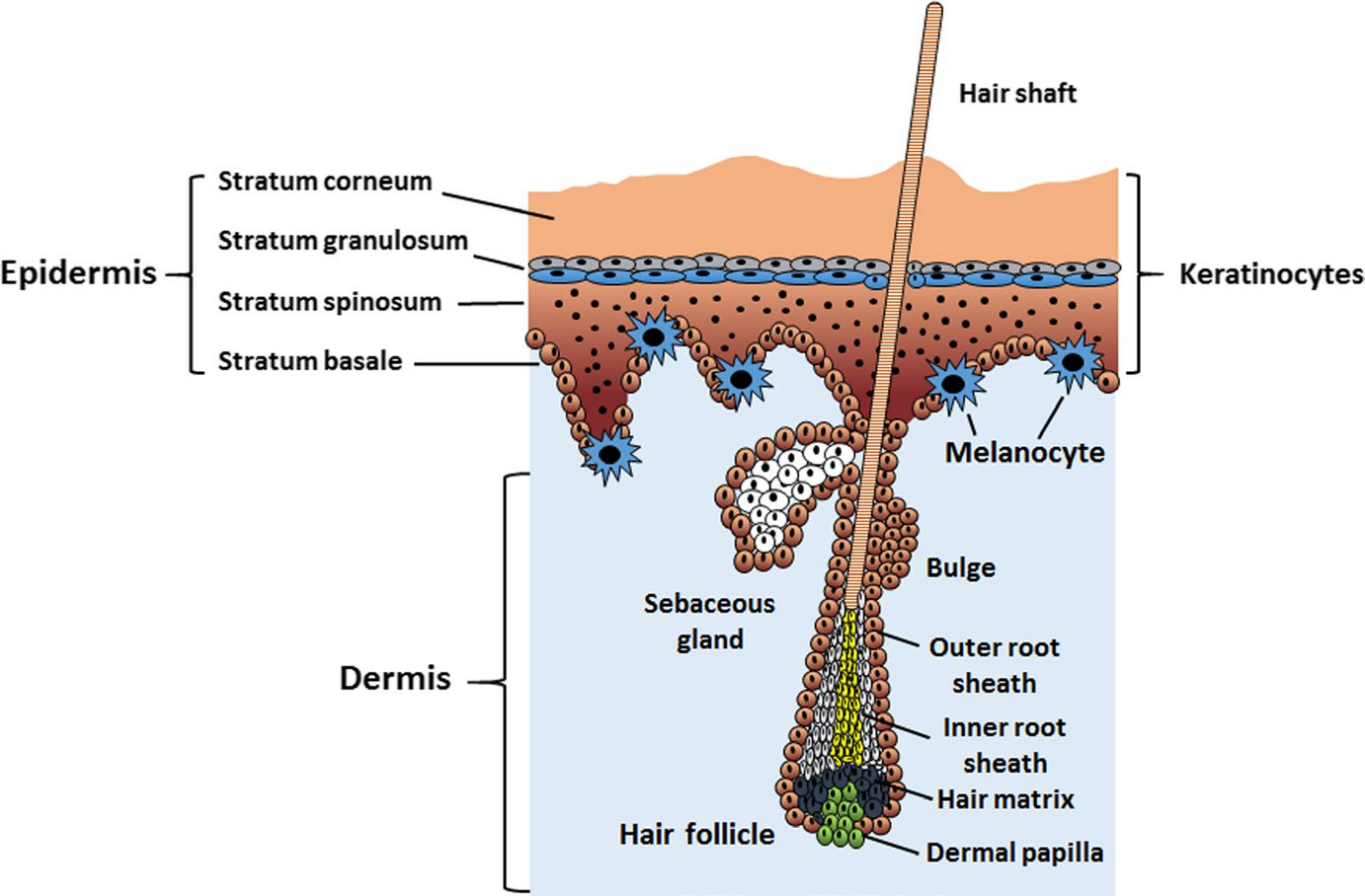
The structure of the epidermis and dermis. Epidermal skin encompasses distinct layers of keratinocytes, including the basal layer (stratum basale), the squamous cell layer (stratum spinosum), the granular layer (stratum granulosum) and the cornified layer (stratum corneum). Melanocytes reside in the bottom layer of the epidermis. Additionally, hair follicles extend from the deeper dermal tissue, through the basement membrane and epithelial layer and extend beyond the border of the skin

According to the physiological structural anatomy, the skin consists of three primary layers: epidermis, dermis and hypodermis.^33^, ^34^ The epidermis, the top layer of skin, encompasses distinct layers of keratinocytes: ①the basal layer comprises self‐renewing progenitor cells; ② the spinous layer, lying above the basal layer, contains upward migrating, differentiating keratinocytes; ③ the granular layer includes cells producing the substrates necessary to form the impermeable barrier; and ④ the stratum corneum comprises terminally differentiated enucleated lipid‐embedded corneocytes (see Glossary) that have undergone cornification to form the outer skin surface. In addition, melanocytes, which reside in the bottom layer of the epidermis, produce melanin pigment to induce pigmentation and protect against UV light. The dermis, the middle layer of skin, is populated by macrophages, lymphocytes, mast cells, dendritic cells and fibroblasts. Additionally, hair follicle morphogenesis occurs via interactions between epidermal keratinocytes and dermal fibroblasts (Figure 2).

**Figure 3:**
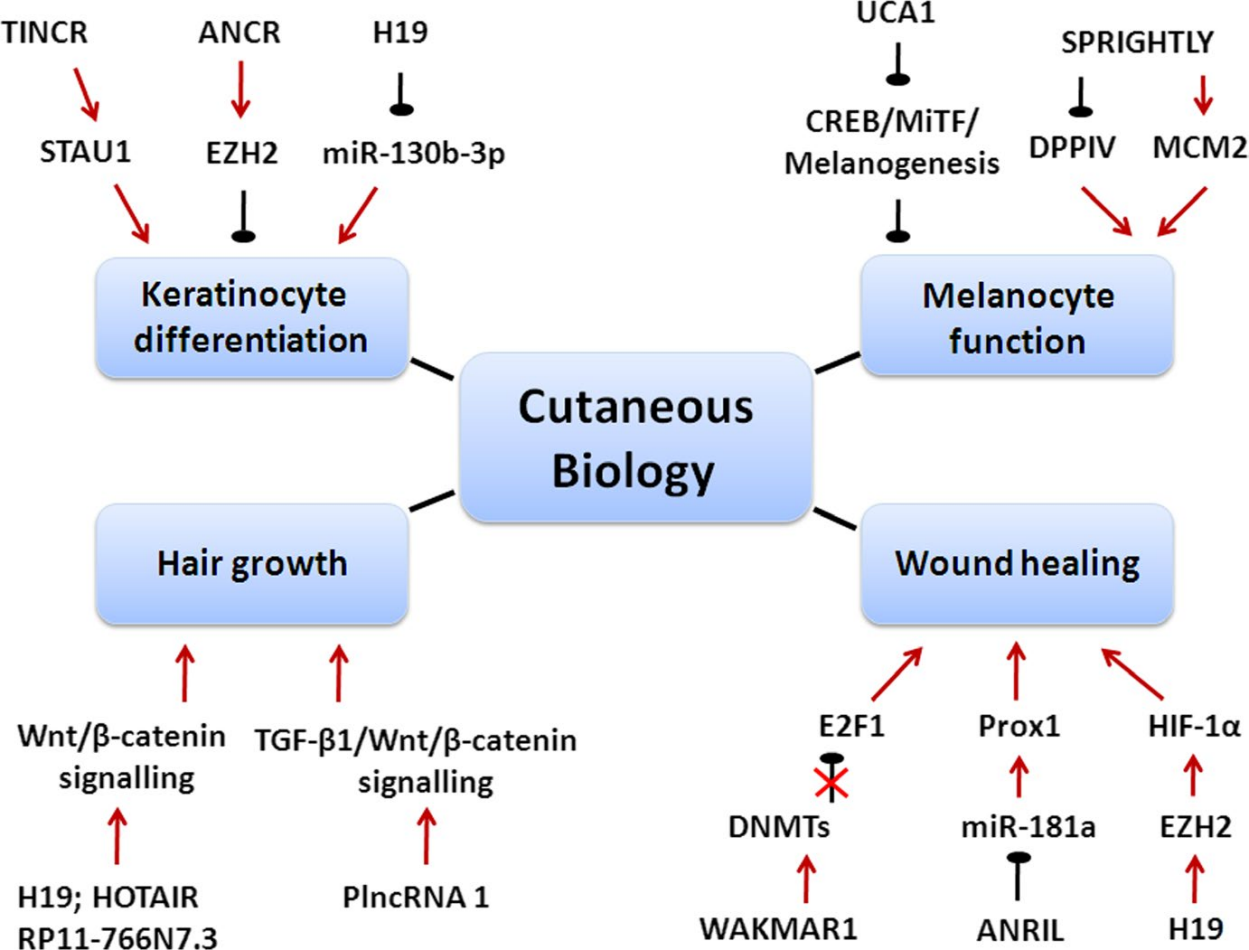
Schematic diagram of functional lncRNAs in cutaneous biology. LncRNAs function as promoters or suppressors in diverse skin physiological processes, including keratinocytes differentiation, melanocyte function, hair growth and wound healing. Red arrow indicates upregulation. Green arrow represents downregulation. Black arrow indicates promotion. 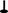 represents suppression. 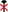 means the abrogation of upstream suppression. ANCR, anti‐differentiation ncRNA; ANRIL, antisense non‐coding RNA in the INK4 locus; CREB, cAMP‐responsive element‐binding protein; DNMTs, DNA methyltransferases; DPPIV, dipeptidyl peptidase IV; E2F1, E2F transcription factor 1; EZH2, enhancer of zeste homolog 2; HIF‐1α, hypoxia‐inducible factor 1 subunit alpha; HOTAIR, HOX transcript antisense RNA; MiTF, microphthalmia‐associated transcription factor; Prox1, Prospero homeobox 1; SPRIGHTLY, SPRY4 intronic transcript 1; STAU1, staufen double‐stranded RNA binding protein 1; TGF‐β1, transforming growth factor beta 1; TINCR, terminal differentiation–induced ncRNA; UCA1, urothelial cancer associated 1; WAKMAR1, wound and keratinocyte migration–associated lncRNA 1

Previous studies majorly focused on the modulatory effects of microRNAs on the processes of keratinocytes' proliferation^35^, ^36^ and differentiation,^37^, ^38^, ^39^ melanocytes biology,^40^, ^41^, ^42^ hair growth^43^, ^44^ and wound healing.^45^, ^46^ In recent years, emerging evidence has indicated that lncRNAs, another kind of non‐coding RNAs, are also involved in these physiological processes (Figure 3；Table 1).

**Table 1:** Functional lncRNAs in cutaneous biology

LncRNAs	Class	Length (bp)	Location	Functions	Targets	Ref.
TINCR	Intronic	3733	Nucleus and Cytoplasm	Keratinocyte differentiation	STAU1	48
ANCR	Intergenic	855	Cytoplasm	Keratinocyte differentiation	EZH2/PRC2	49
H19	Intergenic	2322	Nucleus	Keratinocyte differentiation	miR 130b 3p‐Dsg1	50
SPRIGHTLY	Intronic	703	Cytoplasm	Melanocyte proliferation	MCM2 DPPIV	53
UCA1	Intergenic	2314	Cytoplasm	Melanogenesis	CREB/MiTF	54
PlncRNA 1	Antisense	743	Cytoplasm	Hair growth	TGF‐β1‐Wnt/β‐catenin	56
H19	Intergenic	2322	Nucleus	Hair follicle induction	Wnt/β‐catenin	57
RP11‐766N7.3	Intergenic	557	Nucleus	Hair follicle induction	Wnt/β‐catenin	57
HOTAIR	Antisense	2364	Nucleus and Cytoplasm	Hair follicle induction	Wnt/β‐catenin	57
WAKMAR1	Unclassified	20 700	Nucleus	Wound healing	DNMTs/E2F1	60
H19	Intergenic	2322	Nucleus	Wound healing	EZH2/HIF‐1α	61
ANRIL	Antisense	2659	Nucleus and Cytoplasm	Wound healing	miR‐181a/Prox1	62

### 3.1 LncRNAs regulate keratinocyte differentiation

The epidermis is a stratified surface epithelium that provides a barrier to the external environment. Physiologically, the human epidermis continuously renews itself approximately every 4 weeks by a process of keratinocyte migration, proliferation and differentiation. During the course of epidermal differentiation, a subset of basal keratinocytes withdraws from the cell cycle, detaches from the basement membrane, moves outward from the basal membrane, migrates through the epidermis and undergoes terminal differentiation. A precise balance between the progenitor compartment and terminally differentiated layers is of great importance for the maintenance of the functional epidermis.

To determine the effects of lncRNA on the differentiation of keratinocyte, studies profiled lncRNA expression by microarray and validated the results by qRT‐PCR in the differentiating human keratinocytes using a three‐dimensional epidermal equivalent. In this model, lncRNA BC020554 was found to be downregulated upon keratinocyte differentiation. In contrast, lncRNA AK022798 was upregulated for early keratinocyte differentiation.^47^ However, the biological functions of these differential lncRNAs remain to be elucidated.

Terminal differentiation–induced ncRNA (TINCR), a 3.7 kb nuclear and cytoplasmic intronic lncRNA, was found to control the human epidermal differentiation in vitro by a post‐transcriptional mechanism in differentiated keratinocytes.^48^ Research has revealed that TINCR interacted with a range of differentiation mRNAs through a 25‐nucleotide “TINCR box” motif, which can better help them bind to the staufen double‐stranded RNA binding protein 1 (STAU1) protein, increasing their stabilization in differentiated epidermal strata.

Anti‐differentiation ncRNA (ANCR), another well‐characterized 855 bp cytoplasmic intergenic lncRNA, was downregulated during the terminal differentiation of keratinocytes, adipocytes and osteoblasts in organotypic human tissues.^49^ Contrary to TINCR, ANCR repressed keratinocyte differentiation by associating with the methyltransferase enhancer of zeste homolog 2 (EZH2) and acting as a guide for the polycomb repressive complex 2 (PRC2) chromatin‐modifying complex that eventually leads to epigenetic silencing of target gene loci.

LncRNA H19, a 2322 bp nuclear intergenic lncRNA, promoted the keratinocyte differentiation process in primary human keratinocytes from fresh post‐operative skin samples of children.^50^ Mechanically, H19 acted as an endogenous “sponge,” which bound directly to miR‐130b‐3p. This sponge directly decreased the activity of miR‐130b‐3p and consequently increased the expression of miR‐130b‐3p downstream target desmoglein‐1 (Dsg1), resulting in the promotion of keratinocyte differentiation.

Summarily, these results indicate that lncRNAs have both pro‐differentiation (such as TINCR and H19) and anti‐differentiation (such as ANCR) effects on the processes of keratinocyte differentiation via diverse mechanisms. Further studies are needed to explore the precise roles of lncRNAs in regulating keratinocyte differentiation.

### 3.2 LncRNAs modulate melanocyte functions

Melanocytes, which are located in the basal layer, make up 8% of the epidermis. They produce melanin pigment, which determines the colour of the skin and protects against UV radiation.^51^

Previous studies have revealed that some non‐coding RNAs, such as microRNAs, in melanocytes can alter and affect the synthesis of melanin or the development of melanoma under UV stimulation.^41^, ^52^ However, the roles of lncRNAs in modulating melanocyte proliferation and melanogenesis are still unclear. Growing studies in recent years have characterized some important lncRNAs in controlling melanocyte cell proliferation and melanin pigment production.

As demonstrated, lncRNA SPRIGHTLY (SPRY4 intronic transcript 1, SPRY4‐IT1), a 703 bp cytoplasm‐localized intronic lncRNA, increased human melanocyte proliferation, invasion and colony formation, and induced a multinucleated dendritic‐like phenotype.^53^ Using RNA‐Seq and mass spectrometric analysis of SPRIGHTLY‐expressing cells, researchers have revealed that the aberrant expression of SPRIGHTLY in melanocytes led to the upregulation of cell proliferation genes (such as MCM2) and downregulation of pro‐apoptotic gene (such as dipeptidyl peptidase IV). The findings provide direct evidence for the melanomagenic role of SPRIGHTLY and how it regulates cell proliferation in human melanocytes.

Recently, researchers indicated that lncRNA urothelial cancer associated 1 (UCA1), a 2314 bp cytoplasmic intergenic lncRNA, inhibited melanogenesis in human immortalized melanocytes (PIG1) and human cutaneous melanocytes (MC).^54^ Additionally, UCA1 can also antagonize UVB‐induced pigmentation in PIG1 and MC cells. Mechanically, UCA1 negatively modulated the CREB (cAMP‐responsive element‐binding protein)‐MiTF (microphthalmia‐associated transcription factor)‐melanogenesis axis through inhibiting the cAMP/PKA, ERK and JNK signalling pathways in melanocytes.

Together, these results indicate that lncRNA can modulate melanocyte functions by promoting their proliferation (lncRNA SPRIGHTLY) and reducing their melanogenesis (lncRNA UCA1).

### 3.3 LncRNAs affect hair growth

Hair follicles are one of the important skin appendages located in the dermal layer. Hair structure is complicated, containing an outer root sheath, an inner root sheath and a hair shaft. Hair follicle stem cells (HFSCs), which reside in a specialized region of the outer root sheath designated the bulge, are a vital cell resource of hair follicles and the epidermis.^55^ The differentiation and proliferation of HFSCs in hair follicles are critical for normal hair homeostasis.

lncRNA PlncRNA‐1 (also referred to as CBR3‐AS1), a 743 bp cytoplasmic lncRNA, promoted the proliferation and differentiation of human hair follicle stem cells (HFSCs) through upregulation of TGF‐β1‐mediated Wnt/β‐catenin signalling pathway.^56^ Moreover, compared with early‐passage (passage‐4) dermal papilla (DP) cells from human scalp follicles, lncRNA H19, RP11‐766N7.3 and HOTAIR were aberrantly expressed in late‐passage (passage‐10) DP cells in vitro, which resulted in reducing hair follicle reconstruction via inhibiting Wnt/β‐catenin signalling pathway.^57^

Collectively, these studies show that lncRNAs contribute to key processes underlying hair growth, including the proliferation and differentiation of HFSCs (lncRNA PlncRNA‐1) and hair follicle reconstruction (lncRNA H19, RP11‐766N7.3 and HOTAIR) in DP cells.

### 3.4 LncRNAs influence wound healing and cell proliferation

Wound healing is a fundamental and physiological process required to recover the integrity of the skin after injury, which is achieved through a series of dynamic and complicated processes including inflammation, angiogenesis, coagulation, tissue formation and remodelling.^58^ Failure of these reparative processes leads to chronic impaired wounds, which often happen in patients with underlying disorders, such as diabetes mellitus.^59^ However, the processes of physiological wound healing are intricate. In addition, efficient targeted treatments for chronic impaired wounds are still lacking. It is urgently needed to further explore the underlying molecular mechanism of physiological and pathological wound healing.

LOC105372576, which was also termed wound and keratinocyte migration–associated lncRNA 1 (WAKMAR1), was a nuclear‐localized, critical pro‐migratory lncRNA in human wound‐edge keratinocytes in vitro and human wounds ex vivo.^60^ Mechanistically, it exerted its pro‐migratory functions through activation of E2F1 (E2F transcription factor 1) expression by sequestering DNMTs (DNA methyltransferases) and inhibiting methylation of E2F1 promoter. These findings identify WAKMAR1 as a DNMT‐associated lncRNA in promoting keratinocyte motility and re‐epithelialization, providing human‐specific mechanistic insights into skin wound healing.

lncRNA H19, which was increased in diabetic mouse when the whole blood was preserved by modified preservative fluid, promoted mice fibroblast activation and proliferation to improve wound healing.^61^ Mechanically, H19 could bind to HIF‐1α (hypoxia‐inducible factor 1 subunit alpha) gene promoter region and increase its expression by recruiting EZH2‐mediated histone methylation.

Antisense non‐coding RNA in the INK4 locus (ANRIL), a 2659 bp nuclear and perinuclear cytoplasmic antisense lncRNA, was downregulated in the diabetic wound healing mouse model and high glucose‐induced human lymphatic endothelial cells.^62^ Further functional researches indicated that ANRIL could promote lymphangiogenesis during the diabetic wound healing process via sponging miR‐181a to enhance Prox1 (Prospero homeobox 1) expression.

Together, these results indicated that lncRNAs play an essential functional role in human skin wound healing. Moreover, they also exerted encouraging effects on accelerating the impaired wound healing process in diabetes.

## 4 THE CRITICAL ROLE OF LncRNAS IN CUTANEOUS PROLIFERATION‐RELATED DISEASES

**Figure 4:**
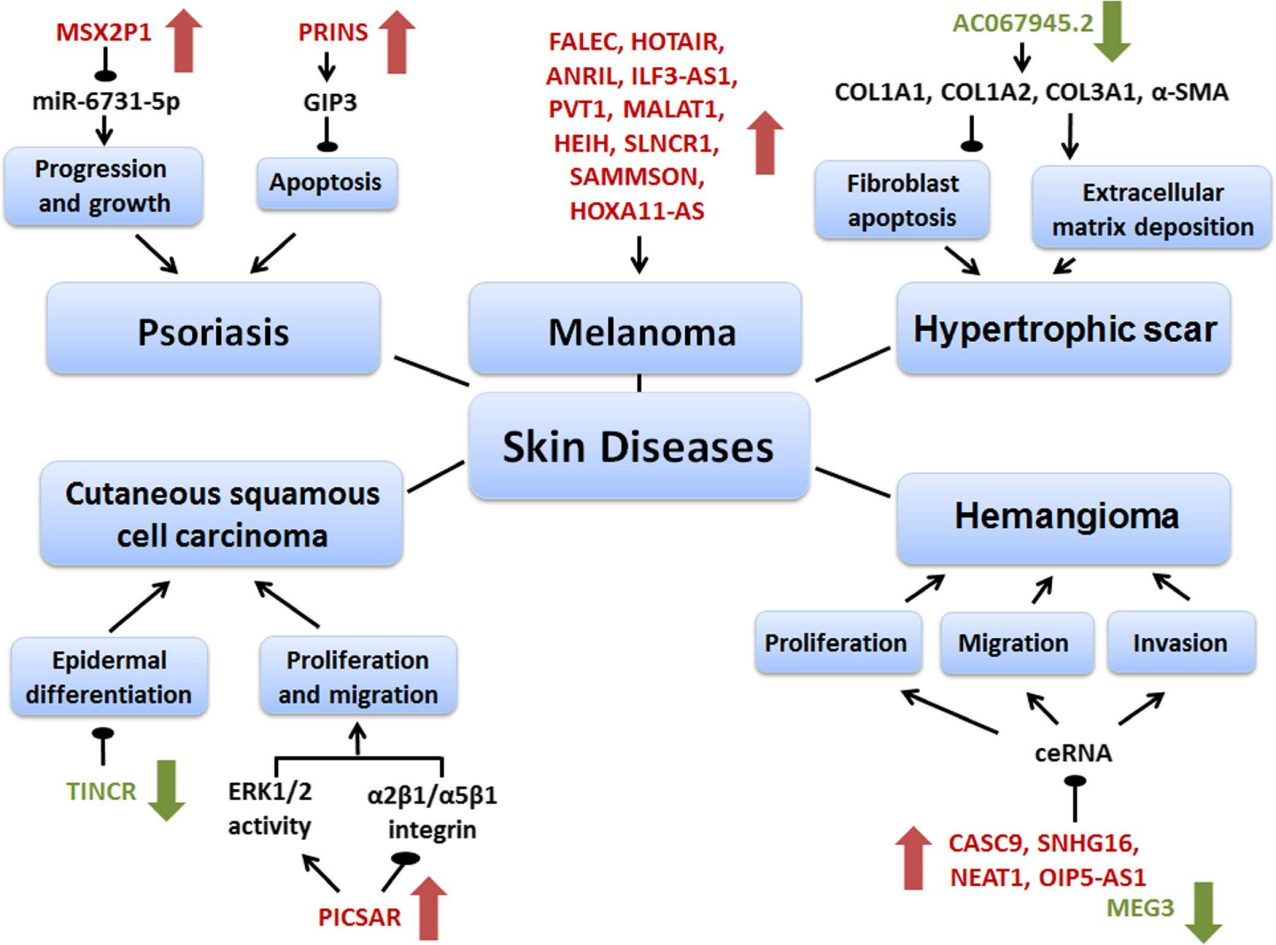
Schematic diagram of dysfunctional lncRNAs in proliferative skin diseases. Dysfunctional lncRNAs play pathogenic roles in cutaneous disorder, such as cutaneous squamous cell carcinoma, psoriasis, melanoma, hypertrophic scar and haemangioma. Red arrow indicates upregulation. Green arrow represents downregulation. Black arrow indicates promotion. 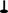 represents suppression. ANRIL, antisense non‐coding RNA in the INK4 locus; CASC9, cancer susceptibility candidate 9; ceRNA, competitive endogenous RNA; FALEC, focally amplified lncRNA on chromosome 1; G1P3, interferon alpha‐inducible protein 6 (IFI6); HOTAIR, HOX transcript antisense RNA; HOXA11‐AS, HOXA11 antisense RNA; MALAT1, metastasis‐associated lung adenocarcinoma transcript 1; MEG3, maternally expressed gene 3; MSX2P1, Msh homeobox 2 pseudogene 1; NEAT1, nuclear‐enriched abundant transcript 1; PICSAR, p38‐inhibited cutaneous squamous cell carcinoma–associated lincRNA; PRINS, psoriasis susceptibility–related RNA gene induced by stress; PVT1, plasmacytoma variant translocation 1; SAMMSON, Survival‐associated mitochondrial melanoma–specific oncogenic non‐coding RNA; SLNCR1, SRA‐like non‐coding RNA; SNHG16, snoRNA host gene 16; TINCR, terminal differentiation–induced ncRNA

Recently, dysfunctional lncRNAs, which result in aberrant keratinocyte differentiation and disturbances of epidermal homeostasis, have also been implicated in the pathogenesis of several hyperproliferative skin diseases, such as cutaneous squamous cancer, melanoma, psoriasis, hypertrophic scar and haemangioma (Figure 4).

### 4.1 LncRNAs in cutaneous squamous cell carcinoma

Cutaneous squamous cell carcinoma (cSCC) is a malignant neoplasm of the skin characterized by an aberrant proliferation of keratinocytes. It is the second‐most common metastatic skin cancer, with a worldwide increasing incidence. Understanding the potential pathology of cSCC will aid in development of effective treatments for cSCC.

To understand the role of lncRNAs in cSCC, Schapoor Hessam and his team performed a human‐related lncRNA microarray and identified 1516 significantly upregulated and 2586 downregulated lncRNAs in cSCC biopsies comparing with non‐lesional epithelial skin.^63^ These results can serve as a template for further, larger functional, in‐depth analyses regarding cSCC‐associated lncRNAs. In addition, TINCR, which was identified as an epidermal differentiation–related lncRNA, was downregulated in human squamous cell carcinoma specimens.^48^ A new study from Minna Piipponen provided evidence that LINC00162, which was also named p38‐inhibited cutaneous squamous cell carcinoma–associated lincRNA (PICSAR), was a specifically upregulated cytoplasmic intergenic lncRNA in primary and metastatic cSCC cell lines in vitro and cSCC tumour cells in vivo. It promoted the proliferation and migration of cSCC cells by activation of ERK1/2 signalling pathway as well as downregulation of α2β1 and α5β1 integrin.^64^, ^65^

In summary, these findings improve the current knowledge that lncRNAs might serve as important mechanistic drivers in cSCC. Beyond this, these works also show significant potentials for the diagnosis and treatment of cSCCs by targeting dysfunctional lncRNAs.

### 4.2 LncRNAs in melanoma

Melanoma is the most lethal cutaneous cancer, with rapid progression and high metastasis potential and recurrence around the world. It is very urgent to develop novel therapeutic strategy by figuring out the underlying pathogenesis of melanoma. Recently, thanks to the advanced developments of biological technology, several lncRNAs have been identified to play vital roles in melanomagenesis (Table 2).

**Table 2:** Dysfunctional lncRNAs in melanoma

LncRNAs	Class	Length (bp)	Location	Functions	Targets	Ref.
ANRIL	Antisense	2659	Nucleus and Cytoplasm	Colony formation	INK4A	66
				Migration	INK4B	67
FALEC	Intergenic	566	Nucleus	Proliferation	EZH2/P21	68
SAMMSON	Intergenic	2027	Cytoplasm	Proliferation	p32	69
HOTAIR	Antisense	2364	Nucleus and Cytoplasm	Proliferation	miR‐152	71
				Invasion		72
				Migration		73
SLNCR1	Intergenic	2257	Nucleus	Invasion	MMP9	70
HEIH	Intergenic	1681	Nucleus and Cytoplasm	Proliferation Invasion Migration	miR‐200b miR‐200a miR‐429	74
HOXA11‐AS	Antisense	1628	Nucleus	Proliferation Invasion Apoptosis	EZH2/P21 miR‐124	75
ILF3‐AS1	Antisense	2032	Cytoplasm	Proliferation Migration Invasion	EZH2/miR‐200b/a/429	76
MALAT1	Intergenic	8302	Nucleus	Proliferation	miR‐183	77
				Migration	miR‐140	78
				Invasion	miR‐22	79
PVT1	Intergenic	1957	Nucleus and Cytoplasm	Proliferation	miR‐26b	80

Abbreviations: EZH2, enhancer of zeste homolog 2; MMP9, matrix metalloproteinase 9.

Antisense non‐coding RNA in the INK4 locus (ANRIL), a 2659 bp nuclear and perinuclear cytoplasmic antisense lncRNA, is identified to co‐cluster with ARF (also known as p14ARF or cyclin‐dependent kinase inhibitor 2A) in a melanoma‐neural system tumour family.^66^ In another study, ANRIL was shown to be upregulated whereas INK4A and INK4B were downregulated in cutaneous melanoma tissues and melanoma cell lines. Interestingly, knockdown of ANRIL restored INK4A and INK4B expression and inhibited colony formation and migration in vitro and growth of melanoma xenograft in vivo.^67^

Focally amplified lncRNA on chromosome 1 (FALEC), a 566 bp nuclear intergenic lncRNA, served as an oncogenic lncRNA in human melanoma. Functional assays showed that silenced FALEC suppressed the proliferation of melanoma cells, resulting in cell cycle arrest and apoptosis. Mechanically, we discovered that FALEC boosted melanoma progression by epigenetically repressing p21 through recruiting EZH2 to the promoter of p21.^68^

Survival‐associated mitochondrial melanoma–specific oncogenic non‐coding RNA (SAMMSON), a recently annotated 2027 bp cytoplasmic intergenic lncRNA with its coding gene located on chromosome 3p13‐3p14, harboured the melanoma‐specific oncogene MITF. Functional assays showed that exogenous SAMMSON increased the clonogenic potential in melanoma cell lines Mel501 and SK‐MEL‐28, whereas SAMMSON knockdown drastically decreased melanoma cell viability and sensitized melanoma to MAPK‐targeting therapeutics in vitro and in patient‐derived xenograft models. Mechanistically, SAMMSON interacted with p32, a master regulator of mitochondrial homeostasis and metabolism, to increase its mitochondrial targeting and pro‐oncogenic function.^69^ These results indicate that silencing of the lineage addiction oncogene SAMMSON disrupts vital mitochondrial functions in a cancer cell–specific manner. Targeting SAMMSON is therefore expected to deliver highly effective and tissue‐restricted anti‐melanoma therapeutic responses.

SRA‐like non‐coding RNA (SLNCR1), an abundantly expressed nuclear intergenic lncRNA associated with decreased melanoma patient survival, increased melanoma invasion. Using the sensitive technique RNA‐associated transcription factor array (RATA), researchers showed that the brain‐specific homeobox protein 3a (Brn3a) and the androgen receptor (AR) bound within and adjacent to SLNCR1's conserved region, respectively. SLNCR1, AR and Brn3a were specifically required for transcriptional activation of matrix metalloproteinase 9 (MMP9) and increased melanoma invasion.^70^ These observations directly link AR to melanoma invasion, possibly explaining why males experience more melanoma metastases and have an overall lower survival in comparison with females.

HOX transcript antisense RNA (HOTAIR), a 2364 bp nuclear antisense lncRNA, was overexpressed in primary melanoma and matched lymph node metastatic tissues.^71^ Interestingly, HOTAIR was also detected in the serum of selected metastatic patients.^72^ Mechanically, HOTAIR promoted the proliferation, invasion and migration of melanoma cells by acting as a competitive endogenous RNA (ceRNA) for miR‐152‐3p.^73^

High expression in hepatocellular carcinoma long non‐coding RNA (HEIH), a highly expressed nuclear and cytoplasmic intergenic lncRNA in human melanoma lesion and cell lines A375, could promote melanoma cell proliferation, invasion and migration through repression of miR‐200b/a/429 transcription.^74^

HOXA11 antisense RNA (HOXA11‐AS), a 1628 bp nuclear antisense lncRNA, was overexpressed in uveal melanoma (UM) tissues from patients and cell lines OCM‐1A, MUM‐2C, C918 and MUM‐28. It could promote UM cell proliferation and invasion and inhibit apoptosis. Mechanistically, RNA immune precipitation demonstrated that HOXA11‐AS could simultaneously interact with EZH2 to suppress its target p21 protein expression. In addition, HOXA11‐AS also functioned as a molecular sponge for miR‐124. Overexpression of miR‐124 attenuated the proliferation and invasion‐promoting effect of HOXA11‐AS.^75^ Collectively, these findings reveal an oncogenic role for HOXA11‐AS in UM tumorigenesis.

Recently, the study found that lncRNA ILF3‐AS1 (a 2032 bp cytoplasmic antisense lncRNA), which was correlated with poor prognosis of melanoma patients, was upregulated in human melanoma tissues and human melanoma cell lines SK‐MEL‐2, SK‐MEL‐28 and A375. Mechanistically, ILF3‐AS1 promoted melanoma cell proliferation, migration and invasion via repressing miR‐200b/a/429 expression by promoting the binding of EZH2 to their promoter.^76^

MALAT1 (metastasis‐associated lung adenocarcinoma transcript 1), a prominent nuclear‐enriched intergenic lncRNA with 8302 nucleotides in length, is initially identified as a prognostic marker for lung cancer metastasis. Accumulating studies now have demonstrated that MALAT1 promoted human melanoma cell proliferation, invasion and migration by acting as ceRNA. As an endogenous sponge, MALAT1 upregulated integrin β1 by downregulating miR‐183.^77^ In addition, MALAT1 increased Slug and ADAM10 expression by silencing of miR‐140.^78^ Moreover, MALAT1 modulated MMP14 and Snail by sponging miR‐22.^79^

In addition, studies have shown that the expression of lncRNA plasmacytoma variant translocation 1 (PVT1), an intergenic lncRNA diffusing throughout the nucleus and cytoplasm, was significantly upregulated in human melanoma tissues and was associated with poor prognosis. Bioinformatics analysis and dual‐luciferase reporter assays revealed that PVT1 acted as a carcinogenic lncRNA by sponging tumour suppressor miR‐26b.^80^

Taken together, these studies indicate that dysfunctional lncRNAs have oncogenic effects on the pathology of melanoma.

### 4.3 LncRNAs in psoriasis

Psoriasis is a multifactorial, hyperproliferative, chronic inflammatory skin disease that affects 1%‐3% of the world's population. Gene expression changes contribute to abnormal proliferation and differentiation of basal keratinocytes in psoriasis lesions. In addition to genes encoding proteins with characterized functions, emerging evidence indicates that lncRNAs also play a vital role in psoriasis.

At first, psoriasis susceptibility–related RNA gene induced by stress (PRINS) was found to be overexpressed in the uninvolved epidermis of psoriatic patients compared with both psoriatic lesional and healthy epidermis, suggesting a role for PRINS in psoriasis susceptibility. As reported, stress signals, such as UVB irradiation, viral infection (herpes simplex virus) and translational inhibition, can increase the RNA level of PRINS. High levels of PRINS expression in psoriatic non‐lesional keratinocytes led to alteration of the stress response in non‐lesional epidermis and contributed to psoriasis pathogenesis.^81^, ^82^ Mechanistically, PRINS may contribute to psoriasis by decreasing sensitivity to spontaneous keratinocyte apoptosis via the regulation of G1P3.^81^

Due to progress in genome sequencing, high‐throughput complementary RNA‐Seq has been used to reveal the differential expression profiles of lncRNAs in normal and psoriatic skin. First, Sebo Withoff provided us with an overview that many transcripts were enriched in autoimmune and immune‐related disorders (AID). The observed enrichment of lncRNA transcripts in AID loci implies that lncRNAs might play an important role in AID aetiology and suggests that these differential lncRNAs in AID should be studied in more detail correctly to interpret genome‐wide association study (GWAS) findings.^83^

Then, James T Elder and his team used RNA‐Seq to analyse 99 lesional psoriatic, 27 uninvolved psoriatic and 90 normal skin biopsies. They detected 2942 previously annotated and 1080 novel lncRNAs, which were expected to be skin‐specific. Their results indicated that many lncRNAs, in particular those that were differentially expressed, were co‐expressed with genes involved in immune‐related functions. Additionally, novel lncRNAs were enriched in the epidermal differentiation complex. They also identified distinct tissue‐specific expression patterns and epigenetic profiles for novel lncRNAs. Together, these results indicate that great deals of lncRNAs are involved in the immune pathogenesis of psoriasis.^84^

In addition, Wilson Liao and his team characterized the landscape of lncRNAs in healthy (NN) and psoriatic skin. They also studied the lncRNA transcriptome in lesional skin biopsy samples from psoriasis patients before (PP) and after treatment (PT) with adalimumab. This research identified the differential expression of 971 lncRNAs between PP and NN, 157 between PP and PT, and 377 between PT and NN. Liao's findings highlight the potential importance of lncRNAs in the pathology of psoriasis and their response to the treatment with adalimumab.^85^ Additionally, Wilson Liao applied weighted gene co‐expression network analysis (WGCNA) to analyse the RNA‐Seq data from psoriasis patients and healthy controls. Network analysis revealed previously unreported biological pathways and roles for coding genes and lncRNAs in psoriasis.^86^

Recently, researchers have suggested that the cytoplasmic lncRNA Msh homeobox 2 pseudogene 1 (MSX2P1) was upregulated in clinical psoriatic lesions compared with normal healthy skin tissues, human immortalized keratinocyte cells and normal human epidermal keratinocyte cells. LncRNA MSX2P1 facilitated the progression and growth of IL‐22‐stimulated keratinocytes by serving as an endogenous sponge directly binding to miR‐6731‐5p and activating S100A7. We speculate that the biological network of MSX2P1‐miR‐6731‐5p‐S100A7 might be a potential novel therapeutic target for the future treatment of psoriasis.^87^

In summary, these sequencing results show the differential expression profiles of lncRNAs between healthy and psoriatic skin. Moreover, functional studies indicate that lncRNAs are important contributors to key processes in psoriasis. Furthermore, these results also provide a novel basis for the development of diagnostic and treatment options for patients with psoriasis.

### 4.4 LncRNAs in hypertrophic scar

Hypertrophic scar (HS), a pathological response to skin wound healing, is characterized by the invasive growth of fibroblasts and the excessive deposition of collagen.^88^, ^89^ It is a common and inevitable outcome of deep skin trauma or severe burn injury. The overall incidence of hypertrophic scars for skin trauma is 40%‐70%, whereas the incidence of burn scars is as high as 80%.^90^ Hypertrophic scars can significantly affect the health of patients via causing pain, pruritus and contractures. Therefore, a thorough understanding of the pathophysiology of HS may help to improve the treatment strategy for HS. Recently, growing evidence suggests that lncRNAs are involved in the occurrence and development of hypertrophic scar.

AC067945.2 (also called NONHSAT076109 in NONCODEv5), a 623 bp antisense lncRNA, was downregulated by transforming growth factor‐β1 (TGF‐β1) in hypertrophic scar tissues compared to corresponding matched normal skin tissues. Mechanistically, AC067945.2 can significantly promote early apoptosis and reduce the expression of COL1A1, COL1A2, COL3A1 and α‐SMA in normal skin fibroblasts. Therefore, downregulated AC067945.2 in hypertrophic scar tissues contributed to the pathological hypertrophic scar formation via inhibiting fibroblast apoptosis and promoting extracellular matrix deposition.^91^

On the other hand, lncRNAs might be negative regulators to hypertrophic scar. COL1A2‐AS1 (also named lncRNA8975‐1), a 352 bp antisense lncRNA, was overexpressed in hypertrophic scar tissues and corresponding hypertrophic scar fibroblasts. It might serve as a negative modulator by inhibiting fibroblast proliferation and reducing COL1A2, COL1A1, COL3A1 and α‐SMA expression in the process of hypertrophic scar.^92^

Together, lncRNAs may play a double‐sided role in the pathological process of hypertrophic scar. Further studies are needed to elaborate precise effects of lncRNAs on hypertrophic scar.

### 4.5 LncRNAs in haemangioma

Haemangioma (HA), which is the most common benign vascular neoplasm of premature infants and infants with low birthweight, is resulted from abnormal proliferation of endothelial cells.^93^, ^94^ It threatens the lives of young children for its high speed in growth or invasion. Therefore, it is urgent to do more research to figure out the pathogenesis of haemangioma. Current advances in the identification of long non‐coding RNAs and their interaction with their target genes have enhanced our awareness of HA pathogenesis.

CASC9 (cancer susceptibility candidate 9), a 1.4 kb cytoplasmic intergenic lncRNA, was obviously overexpressed in human proliferating phase (rapid growth) HA tissues than that in involuting phase (spontaneous regression) HA tissues and normal tissues.^95^ Further studies indicated that CASC9 accelerated the proliferation, invasion and migration of HA‐derived endothelial cell (HDECs) via negatively regulating miR‐125a‐3p/Nrg1 (neuregulin 1) axis.

OIP5‐AS1, a 1.7 kb cytoplasmic antisense lncRNA, was upregulated in both human involuting and proliferating phase haemangioma tissues.^96^ Accumulating results indicated that OIP5‐AS1 promoted the proliferation of human haemangioma vascular endothelial cells (HemECs) via regulating miR‐195‐5p/NOB1 (NIN1 binding protein 1 homolog) axis.

SNHG16 (snoRNA host gene 16), a 2.4 kb cytoplasm‐localized sense‐overlapping long non‐coding RNA, was obviously higher in human proliferating phase HA tissues than that in involuting phase HA tissues and normal tissues.^97^ Mechanically, upregulated SNHG16 drives proliferation, migration and invasion of HemECs through modulation of miR‐520d‐3p/STAT3 axis.

NEAT1 (nuclear‐enriched abundant transcript 1), a 3.7 kb nuclear paraspeckle‐localized intergenic lncRNA, was elevated in human HA tissues, especially in proliferating phase Has.^98^ Further mechanism demonstrated that NEAT1 facilitated the progression of haemangioma via functioning as a ceRNA for miR‐361‐5p to regulate VEGFA expression.

In addition, lncRNAs were also indicated to function as negative regulators on HA proliferation. LncRNA MEG3 (maternally expressed gene 3), a 1.6 kb intergenic lncRNA, can inhibit human HA tumorigenesis by sponging miR‐494 and regulating PTEN/PI3K/AKT pathway.^99^ However, the expression of MEG3 was obviously downregulated in proliferating phase human HAs, which eventually promoted the development of HAs.

In summary, these studies indicate that dysfunctional lncRNAs might be vital contributors to the pathology of haemangioma.

## 5 POTENTIAL DIAGNOSTIC AND THERAPEUTIC APPLICATIONS OF LncRNAS

Advances in the identification and characterization of long non‐coding RNA in dermatological research enhance our understanding of the significance of regulatory non‐coding RNAs in skin development and homeostasis and their implication in regulation of a variety of skin pathological conditions. The tissue‐ or disease‐specific expression of lncRNAs makes them ideal biomarkers.

### 5.1 Biomarkers for the diagnosis and prognosis of skin diseases

The tissue‐ and disease‐specific expression of lncRNAs makes them ideal biomarkers for diagnosis. For example, PICSAR is proved to be specifically expressed by tumour cells in actinic keratosis (UV‐induced premalignant lesions), cSCC in situ and invasive cSCCs tissues but not by keratinocytes in normal skin in vivo, suggesting PICSAR as a specific biomarker for early diagnose of primary and invasive cSCC.^65^ Besides their potential diagnostic implications, lncRNAs could also be used as molecular marker to predict prognosis of cutaneous disorders. Dysfunctional lncRNAs, such as FALEC, HEIH, SLNCR1 and HOTAIR, predict poor outcome in melanoma patients.^68^, ^70^, ^73^, ^74^ Additionally, HOTAIR can also be identified in the serum. The identification of lncRNAs in the blood might suggest its use as a non‐invasive circulating marker for diagnosing cutaneous diseases or as a marker of relapses during the follow‐up.^72^ Moreover, lncRNAs can also act as biomarkers to predict the pathological stages of benign skin diseases. For example, the high level of lncRNA CASC9, SNHG16 and NEAT1 in haemangioma tissues predicts that haemangioma is on the proliferating phase rather than involuting phase. As the Liao's findings, the differential lncRNAs can also be used as biomarkers to monitor and predict the therapeutic responses to the treatment with adalimumab.^84^ Overall, these results indicate that lncRNAs could serve as particularly useful biomarkers for diagnosis and prognosis of cutaneous disorders (Table 3).

**Table 3:** Skin disease–related lncRNAs as diagnostic and prognostic biomarkers

LncRNAs	Diagnostic	Strategy	Prognostic	Ref.
PICSAR	Upregulation in primary and metastatic human cSCC in vitro and in vivo	RNA‐Seq, qRT‐PCR	Unknown	64, 65
FALEC	Upregulation in human melanoma tissues and cell lines	qRT‐PCR	Upregulation associated with invasion, distal metastasis potentials and poor survival in melanoma	68
SLNCR1	Upregulation in human melanoma tissues and cell lines	RNA‐Seq, qRT‐PCR	Upregulation associated with high severity and poor survival in melanoma	70
HOTAIR	Upregulation in primary and metastatic human melanoma	qRT‐PCR, GEO analysis	Upregulation associated with metastases potentials and poor survival in melanoma	71, 72, 73
HEIH	Upregulation in human melanoma tissues	qRT‐PCR	Upregulation associated with advanced clinical stages and poor survival in melanoma	74
ILF3‐AS1	Upregulation in primary and metastatic human melanoma	Database analysis, qRT‐PCR	Upregulation associated with metastatic potenials and poor prognosis in melanoma	76
PVT1	Upregulation in human melanoma tissues	qRT‐PCR	Upregulation associated with poor prognosis in melanoma	80
PRINS	Upregulation in human psoriatic non‐lesional epidermis	cDNA library, qRT‐PCR	Associated with psoriasis susceptibility	81, 82
MSX2P1	Upregulation in human psoriatic lesions	Microarray, qRT‐PCR	Unknown	87
AC067945.2	Downregulated in human hypertrophic scar tissues	qRT‐PCR	Downregulation associated with the development of hypertrophic scar	91
COL1A2‐AS1	Upregulation in human hypertrophic scar tissue and hypertrophic scar fibroblasts	qRT‐PCR	Unknown	92
CASC9	Obvious upregulation in human proliferating phase haemangioma (HA)	qRT‐PCR	Upregulation associated with the development of HA	95
OIP5‐AS1	Upregulation in both human involuting and proliferating phase HA	qRT‐PCR	Upregulation associated with the development of HA	96
SNHG16	Obvious upregulation in human proliferating phase HA	qRT‐PCR	Upregulation associated with the development of HA	97
NEAT1	Obvious Upregulation in human proliferating phase HA	qRT‐PCR	Upregulation associated with the development of HA	98
MEG3	Downregulation in the human proliferating phase HA	qRT‐PCR	Downregulation associated with the development of HA	99

Abbreviations: cSCC, cutaneous squamous cell carcinoma; HA, haemangioma; RNA‐Seq, RNA sequencing.

### 5.2 Therapeutic strategies for skin diseases

Given the importance of lncRNA in skin diseases, lncRNAs might be promising therapeutic targets for treatments. Recent research has demonstrated that lncRNAs can be knocked down via the utilization of RNA interference (RNAi).^100^, ^101^ Most recently, utilizing or developing new and cutting‐edge technologies, such as antisense oligonucleotide (ASO),^102^, ^103^ locked nucleic acid (LNA)^69^, ^104^ or bridged nucleic acid (BNA) technologies,^105^ and CRISPR‐mediated gene editing,^106^, ^107^, ^108^ will aid in targeting the dysfunctional lncRNAs for their therapeutic values. Excitingly, targeting the lncRNA MALAT1 via LNA gapmeR ASO has recently been indicated to trigger anti‐multiple myeloma activities, providing proof of concept that the therapeutic potential of targeting lncRNAs.^109^ Thus, targeting lncRNAs will be a promising therapeutic strategy for skin diseases.

## 6 CONCLUSIONS AND FUTURE PERSPECTIVE

Cutaneous biology and skin disease–related lncRNAs are still an emerging field, with only a few of them being characterized for their biological functions and clinical implications. This review summarizes the current knowledge regarding the promotive or inhibitory regulations of lncRNAs to skin development and epidermal homeostasis. Besides, this review also describes the pathogenic role of dysfunctional lncRNAs in hyperproliferative skin diseases.

However, there are many mysteries remaining, including whether the evolution of lncRNAs is conserved. Given that lncRNAs within distinct model animals do not show strict homology, future studies need to focus on the in vivo biological functions of cutaneous lncRNAs.

In addition, the functional sequences and domains that execute the functions of lncRNAs remain to be fully investigated. In future, using artificial intelligence or neural network,^110^, ^111^ researchers may precisely predict the binding between lncRNAs and proteins. Moreover, with the development of cross‐linking methods, such as capture hybridization analysis of RNA targets (Chart),^112^ photoactivatable ribonucleoside‐enhanced cross‐linking and immunoprecipitation (PAR‐CLIP)^113^ and individual nucleotide resolution UV cross‐linking and immunoprecipitation (iCLIP),^114^ researchers can map the network of interactions that the lncRNA establishes with DNA, RNA and proteins. Furthermore, structure‐related technologies, such as selective 2′‐hydroxyl acylation analysed by primer extension (SHAPE),^115^, ^116^ will provide detailed evidences to understand structure‐function interrelationships for lncRNAs.

There are also some challenges of using lncRNAs as biomarkers. For instance, the amount of lncRNAs in plasma is generally low. Therefore, unlike short non‐coding RNAs, such as miRNAs, most lncRNAs are not detectable in plasma by standard methods, such as microarrays or quantitative PCR. More studies are required to examine whether lncRNAs are better predictive biomarkers than protein‐coding genes or other non‐coding RNAs (such as miRNAs).

Additionally, the full potential of using lncRNAs in skin disease therapy has not yet been fully explored now. Better understanding of precise biological functions of lncRNAs and better targeting technologies are required, which will advance the lncRNA therapy. In future, more and more clinical trials targeting various lncRNAs are ongoing for the treatment with cutaneous diseases.

## CONFLICT OF INTEREST

The authors have declared no conflicting interests.

## AUTHOR CONTRIBUTIONS

LT, YL and HX wrote the manuscript; YL, XY and HX searched the literature; LT and GZ edited the paper.

## Data Availability

The data that support the findings of this study are available from the corresponding author upon reasonable request.
